# Publisher Correction: Coherent modulation of the sea-level annual cycle in the United States by Atlantic Rossby waves

**DOI:** 10.1038/s41467-018-06852-4

**Published:** 2018-10-12

**Authors:** Francisco M. Calafat, Thomas Wahl, Fredrik Lindsten, Joanne Williams, Eleanor Frajka-Williams

**Affiliations:** 1 National Oceanography Centre, Joseph Proudman Building, 6 Brownlow Street, L3 5DA Liverpool, UK; 2 Department of Civil, Environmental and Construction Engineering and National Center for Integrated Coastal Research, University of Central Florida, 12800 Pegasus Drive, Suite 211, Orlando, FL USA; 3 Department of Information Technology, Uppsala University, Lägerhyddsv. 2, hus 2, 752 37, Uppsala, Sweden; 4 Ocean and Earth Sciences, University of Southampton, European Way, SO14 3ZH Southampton, UK

Correction to: Nature Communications 10.1038/s41467-018-04898-y; published online 3 July 2018.

The original version of this Article contained an error in the first sentence in the legend of Fig. 1, which incorrectly read ‘The first letter of ‘Hatteras’ should be capitalized, in both Figure 1a and 1b since Hatteras is a proper noun.’ The correct version removes this sentence. This has been corrected in both the PDF and HTML versions of the article.

