# Supplementary material for: Coherent modulation of the sea-level annual cycle in the United States by Atlantic Rossby waves
Source: Nat Commun. 2018 Jul 3;9:2571. doi: 10.1038/s41467-018-04898-y (PMC6030228; doi:10.1038/s41467-018-04898-y)
Supplement: Supplementary file 1 — Supplementary Information [file 41467_2018_4898_MOESM1_ESM.pdf]

Coherent modulation of the sea-level annual cycle in the United States by Atlantic Rossby waves

Calafat et al.

Supplementary Figures 1-6.

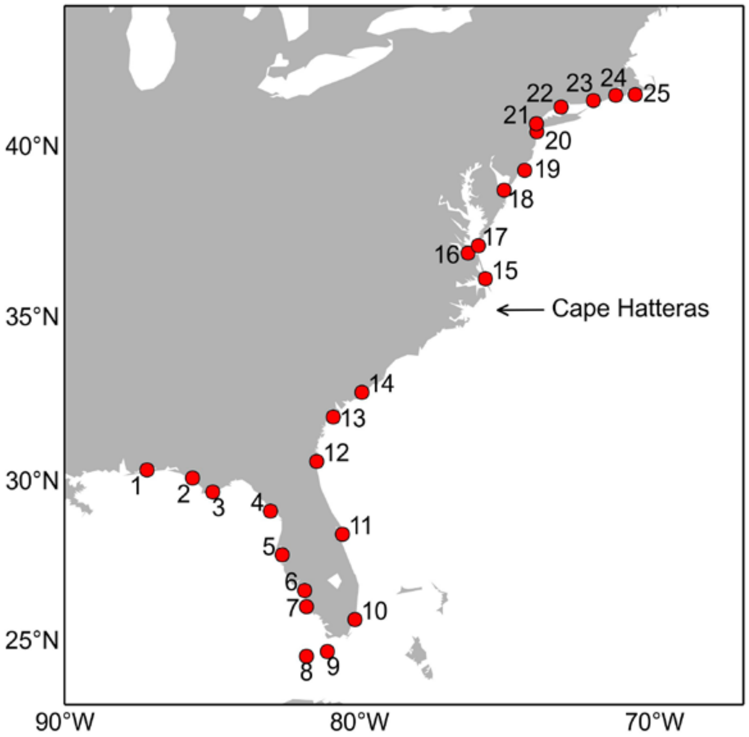

Supplementary Figure 1. Location of the tide gauges used in this study. The tide-gauge identification numbers correspond to those of Fig. 1.

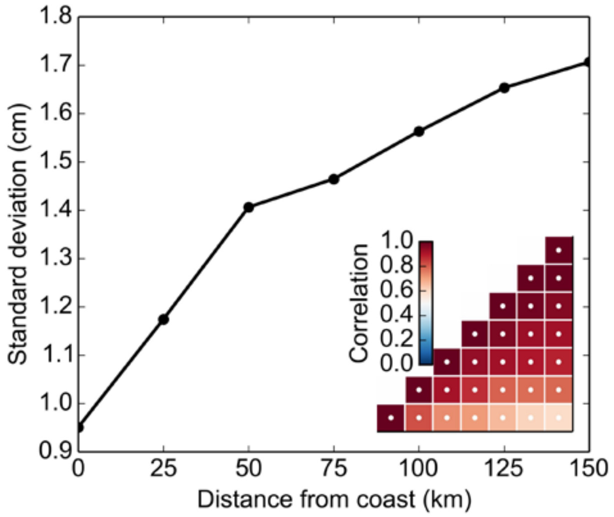

Supplementary Figure 2. The standard deviation of the SLAC modulator as a function of distance from the Bahamas east coast at 26.5°N, together with the cross-correlation between the corresponding time series (inset). In the inset correlation matrix, the coast corresponds to the bottom-left corner and white dots denote significant correlation at the 95% confidence level. To compute correlations we have accounted for a lag due to propagation.

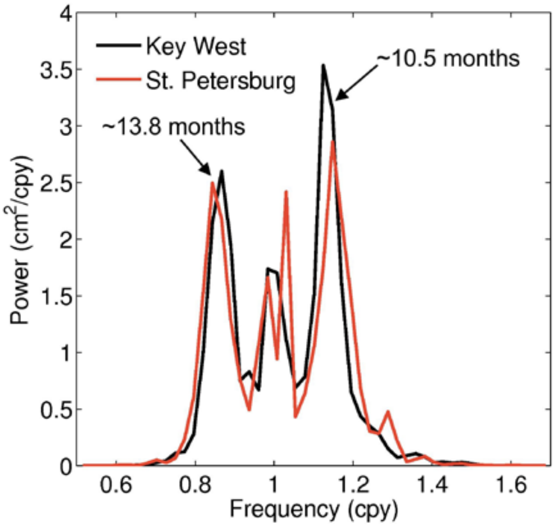

Supplementary Figure 3. Power spectral density of the SLAC modulator for the Key West and St. Petersburg tide gauges. The spectral density estimation has been computed using the Welch's method with a window of 400 months and without overlap.

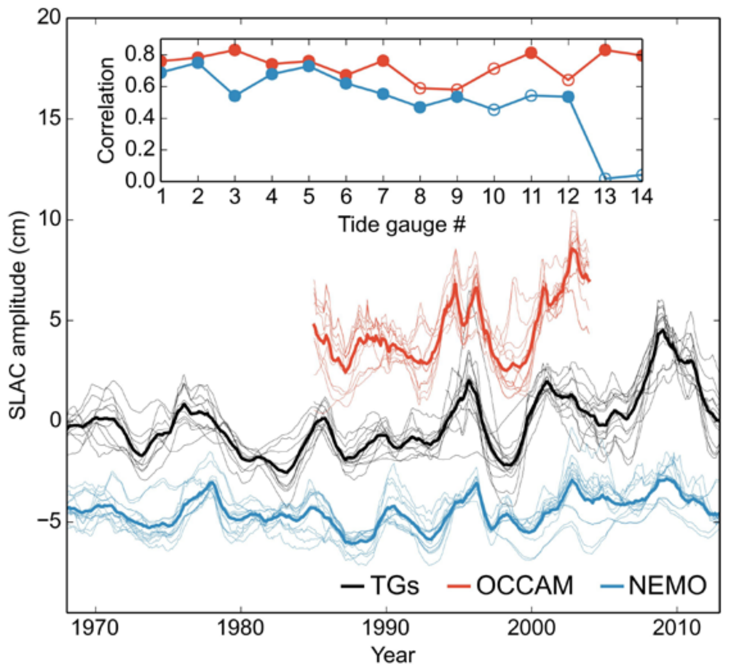

Supplementary Figure 4. Time series of the SLAC amplitude from tide gauges (black), OCCAM (red), and NEMO (blue). Thin curves correspond to time series for individual locations whereas thick curves denote their average. The correlation between the modeled amplitudes and the observations at individual stations is shown in the inset. Filled circles denote significant correlation at the 95% confidence level. The tide-gauge identification numbers correspond to those of Fig. 1.

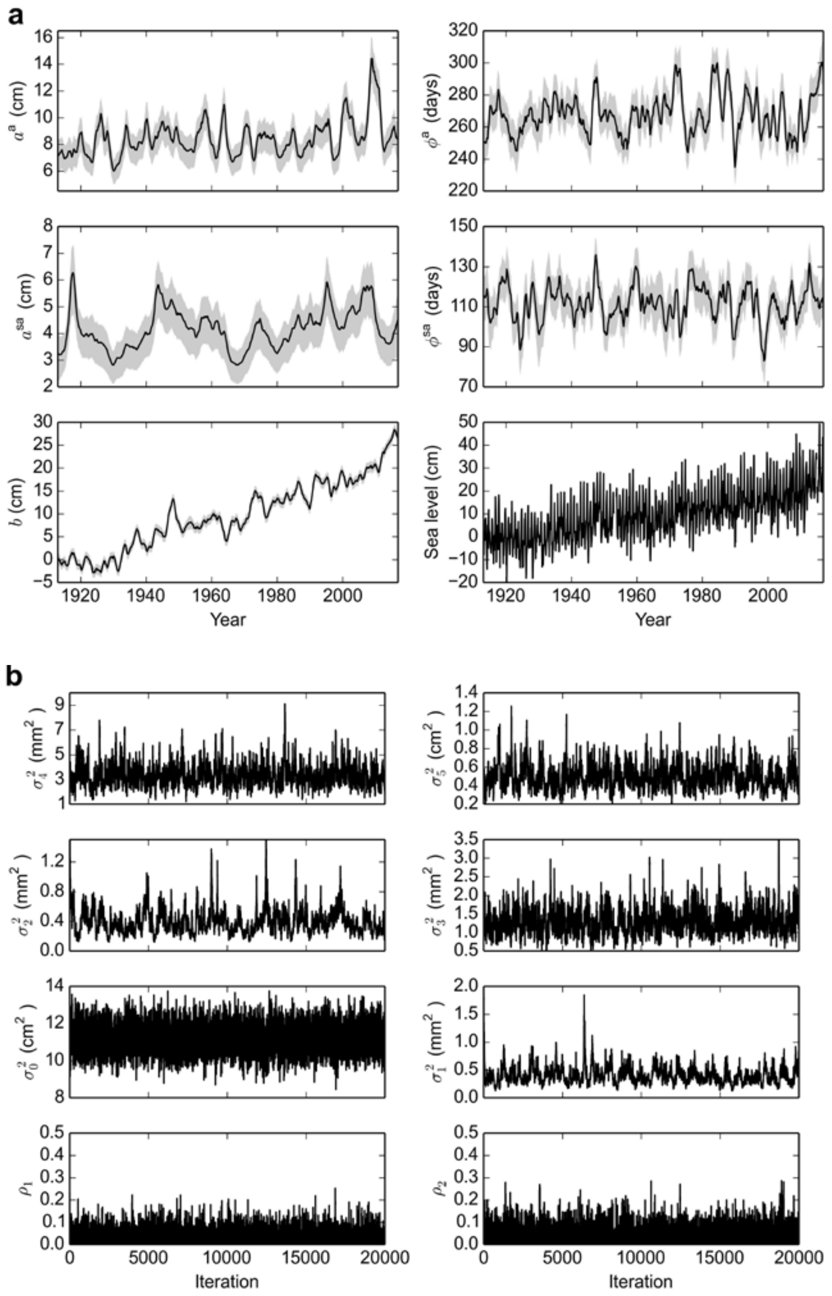

Supplementary Figure 5. a, Instantaneous amplitudes (a^{a} and a^{sa}) and phases (ϕ^{a} and ϕ^{sa}) of the annual and semi-annual cycles, and the low-frequency component (*b*) for the Key West tide gauge as estimated using a Bayesian state-space model. The black curves represent the mean of the posterior distribution at each time step while the grey-shaded area denotes the 68% (1 sigma) credible interval. The sea level from the tide-gauge record is also shown in the bottom-right panel. Note that in this plot ϕ^{a} and ϕ^{sa} represent the so-called phase offset rather than the phase. **b**, trace plots for the static parameters of the state-space model, $\theta = (\rho_1, \rho_2, \sigma_0^2, \sigma_1^2, \sigma_2^2, \sigma_3^2, \sigma_4^2, \sigma_5^2)$, as estimated by PGAS for Key West.

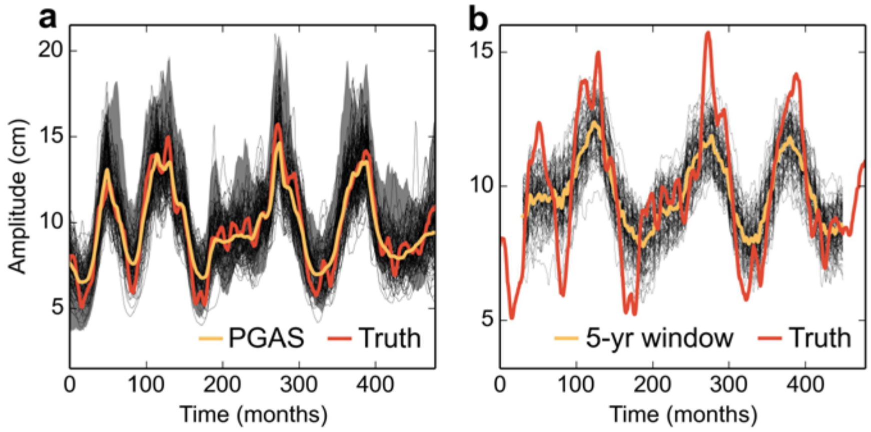

Supplementary Figure 6. a, synthetic time series of the annual amplitude (red) along with estimates computed using a Bayesian state-space model for 100 different realizations of the noise (black lines). The orange curve is the mean of the 100 estimates whereas the grey-shaded area denotes the 95% credible interval for one of the 100 estimates. **b**, the same as **a** but for estimates obtained using a harmonic fit to 5-yr running windows.
